# Impact of the distal resection margin on local recurrence after neoadjuvant chemoradiation and rectal excision for locally advanced rectal cancer

**DOI:** 10.1038/s41598-021-02438-1

**Published:** 2021-11-25

**Authors:** Seung Ho Song, Jun Seok Park, Gyu-Seog Choi, An Na Seo, Soo Yeun Park, Hye Jin Kim, Sung-Min Lee, Ghilsuk Yoon

**Affiliations:** 1 Colorectal Cancer Center, Kyungpook National University Chilgok Hospital, School of Medicine, Kyungpook National University, 807 Hogukro, Buk-gu, Daegu, 40414 Republic of Korea; 2 Department of Pathology, Kyungpook National University Chilgok Hospital, School of Medicine, Kyungpook National University, Daegu, Republic of Korea

**Keywords:** Colorectal cancer, Surgical oncology

## Abstract

We aimed to evaluate whether a short distal resection margin (< 1 cm) was associated with local recurrence in patients with locally advanced rectal cancer who underwent preoperative chemoradiotherapy. Patients with rectal cancer who underwent preoperative chemoradiotherapy followed by curative surgery were divided into two groups based on the distal resection margin (≥ 1 cm and < 1 cm). In total, 507 patients were analyzed. The median follow-up duration was 48.9 months. The 3-year local recurrence rates were 2% and 8% in the ≥ 1 cm and < 1 cm groups, respectively (*P* < 0.001). Multivariable analysis revealed that a distal resection margin of < 1 cm was a significant risk factor for local recurrence (*P* = 0.008). Subgroup analysis revealed that a distal resection margin of < 1 cm was not an independent risk factor for local recurrence in the ypT0–1 group. However, among patients with tumor stages ypT2–4, the cumulative 3-year incidences of local recurrence were 2.3% and 9.8% in the ≥ 1 cm and < 1 cm groups, respectively (*P* = 0.01). A distal resection margin of < 1 cm might influence local recurrence rates in patients with locally advanced rectal cancer undergoing preoperative chemoradiotherapy, especially in patients with tumor stages ypT2–4.

## Introduction

Ensuring an adequate distal resection margin (DRM) is necessary to save the sphincter in patients with locally advanced rectal cancer (LARC)^1, 2^. The National Comprehensive Cancer Network guidelines state that a negative DRM of 1–2 cm might be acceptable^1^, and current Japanese guidelines recommend a DRM of ≥ 2 cm in patients with lower rectal cancers at stages 0–III^2^. However, in routine clinical practice, it is sometimes technically difficult to follow these guidelines for such cases. Occasionally, margins that are thought to be sufficient during the initial surgery have been reported to be shorter than that anticipated. Even among patients receiving preoperative chemoradiotherapy (CRT), unpredictable tumor shrinkage and residual tumor patterns make it difficult to determine an acceptable DRM after surgery^3, 4^.

Various studies have investigated the optimal DRM needed to achieve acceptable oncologic outcomes after sphincter-saving surgery for LARC^5–8^. Although a DRM of < 1 cm might not be associated with specific oncologic outcomes, according to a systematic review^9^, the authors emphasized that the specific rules for patients and tumor selection need to be evaluated. To our knowledge, few studies have evaluated the impact of DRM on local recurrence after preoperative CRT and according to the ypT tumor stage. Herein, we aimed to evaluate whether a short DRM (< 1 cm) was associated with local recurrence in patients with LARC who underwent preoperative CRT. We compared oncologic outcomes according to the DRM and performed subgroup analysis according to the ypT stage.

## Results

### Clinical characteristics

**Figure 1:**
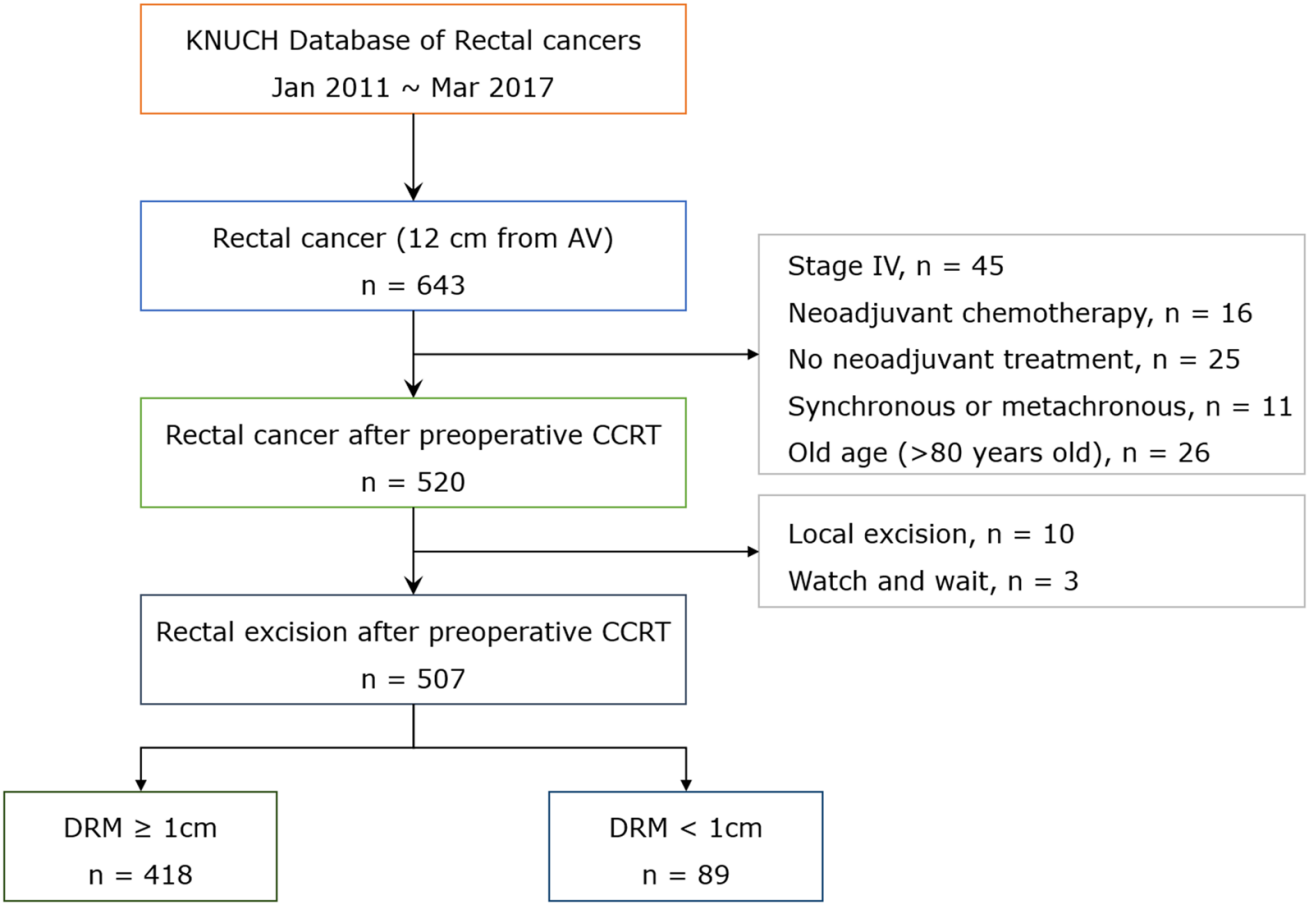
Study flow diagram.

Overall, 507 patients with LARC underwent preoperative CRT and rectal excision (Fig. 1). Patient characteristics are summarized in Table 1. For the DRM dimensions, there were 418 patients in the ≥ 1 cm group and 89 in the < 1 cm group. Tumor height (from the anal verge) was significantly greater in the ≥ 1 cm group than in the < 1 cm group (6.0 cm vs. 2.5 cm; *P* < 0.001). Significantly more patients with advanced clinical T stage tumors were included in the ≥ 1 cm group than in the < 1 cm group (clinical stages T3–4, 99.3% vs. 94%, *P* < 0.001).

**Table 1:** Patient characteristics. DRM, distal resection margin.

	DRM ≥ 1 cm (n = 418)	DRM < 1 cm (n = 89)	*P*
Age, years	62.0 (55.0–71.0)	60.0 (53.0–69.0)	0.16
Sex			0.21
Male	254 (60.8%)	61 (68.5%)	
Female	164 (39.2%)	28 (31.5%)	
ASA classification			0.55
1	233 (55.7%)	53 (59.6%)	
2	181 (43.3%)	36 (40.4%)	
3	4 (1.0%)	-	
Tumor height, cm	6.0 (4.0–8.0)	2.5 (2.0–4.2)	< 0.001
Clinical T stage			< 0.001
T2	3 (0.7%)	5 (5.6%)	
T3	339 (81.1%)	79 (88.8%)	
T4	76 (18.2%)	5 (5.6%)	
T4a/T4b	38/38	1/4	
Clinical N stage			0.22
N0	81 (19.4%)	23 (25.8%)	
N +	337 (80.6%)	66 (74.2%)	

*ASA* American Society of Anesthesiologists.

### Levator ani invasion

In the ≥ 1 cm group, suspicious levator ani invasion was seen in 32 patients on the initial pelvic magnetic resonance imaging (MRI). After CRT, it was observed in 29 patients, and 27 of these patients underwent abdominoperineal resection (APR). In the < 1 cm group, suspicious levator ani invasion was seen in 2 patients before CRT, and after CRT, it was observed in 1 patient. Although levator ani invasion was suspected on post-treatment pelvic MRI in 2 patients in the ≥ 1 cm group and 1 patient in the < 1 cm group, it did not appear to be involved intraoperatively. These patients underwent sphincter-saving surgery and had no recurrence during the study period.

### Operative and pathologic outcomes

Operative and pathologic findings are summarized in Table 2. The most common type of operation was intersphincteric resection in the < 1 cm group (61%). Lateral pelvic lymph node dissection was performed in 72 patients (17.2%) in the ≥ 1 cm group and in 11 patients (12.4%) in the < 1 cm group (*P* = 0.33). Among them, pathologic lateral pelvic lymph node metastasis was noted in 23 patients (31.9%) and 2 patients (18.2%) in the ≥ 1 cm and < 1 cm groups, respectively (*P* = 0.57). Pathologic tumor depth differed significantly between the groups: 70.6% of patients in the ≥ 1 cm group had ypT3–4 disease, while 47% of patients in the < 1 cm group had ypT3 disease. No patients in the < 1 cm group had ypT4 grade tumors. The rate of pathologic nodal positivity was significantly higher in the ≥ 1 cm group than in the < 1 cm group (39.7% vs. 16%; *P* < 0.001). Circumferential resection margin (CRM) positivity rates were similar between the ≥ 1 cm and < 1 cm groups (9.6% vs. 9%; *P* = 1.00). The pathological complete response rate differed significantly between the ≥ 1 cm and < 1 cm groups (12.7% vs. 24%; *P* = 0.01). In addition, good tumor response rates (tumor regression grade [TRG] 3 and 4) differed between the ≥ 1 cm and < 1 cm groups (49.8% vs. 71%; *P* < 0.001).

**Table 2:** Operative and pathologic findings. DRM, distal resection margin.

	DRM ≥ 1 cm (n = 418)	DRM < 1 cm (n = 89)	*P*
Type of operation			< 0.001
LAR	286 (68.4%)	35 (39.3%)	
ISR	105 (25.1%)	54 (60.7%)	
APR	27 (6.5%)	-	
LPND	72 (17.2%)	11 (12.4%)	0.33
Tumor size, cm	3.2 (2.0–4.5)	2.5 (1.8–3.3)	< 0.001
Lymphovascular invasion	32 (7.7%)	3 (3.4%)	0.22
Venous invasion	32 (7.7%)	4 (4.5%)	0.41
Tumor perforation	9 (2.2%)	2 (2.2%)	1.00
Pathologic stage			< 0.001
ypT0N0	45 (10.8%)	21 (23.6%)	
ypT0N +	6 (1.4%)	-	
I	60 (14.4%)	24 (27.0%)	
II	147 (35.2%)	30 (33.7%)	
III	160 (38.3%)	14 (15.7%)	
CRM, positive (≤ 1 mm)	40 (9.6%)	8 (9.0%)	1.00
Pathologic LPN positive	23/72 (31.9%)	2/11 (18.2%)	0.57
Tumor regression grade			0.002
0 (no regression)	6 (1.5%)	2 (2.3%)	
1 (minor regression)	52 (12.9%)	8 (9.2%)	
2 (moderate regression)	144 (35.8%)	15 (17.2%)	
3 (good regression)	149 (37.1%)	41 (47.1%)	
4 (total regression)	51 (12.7%)	21 (24.1%)	

*LAR* low anterior resection, *ISR* intersphincteric resection, *APR* abdominoperineal resection, *LPND* lateral pelvic lymph node dissection, *CRM* circumferential resection margin, *LPN* lateral pelvic lymph node.

### Survival outcomes

**Figure 2:**
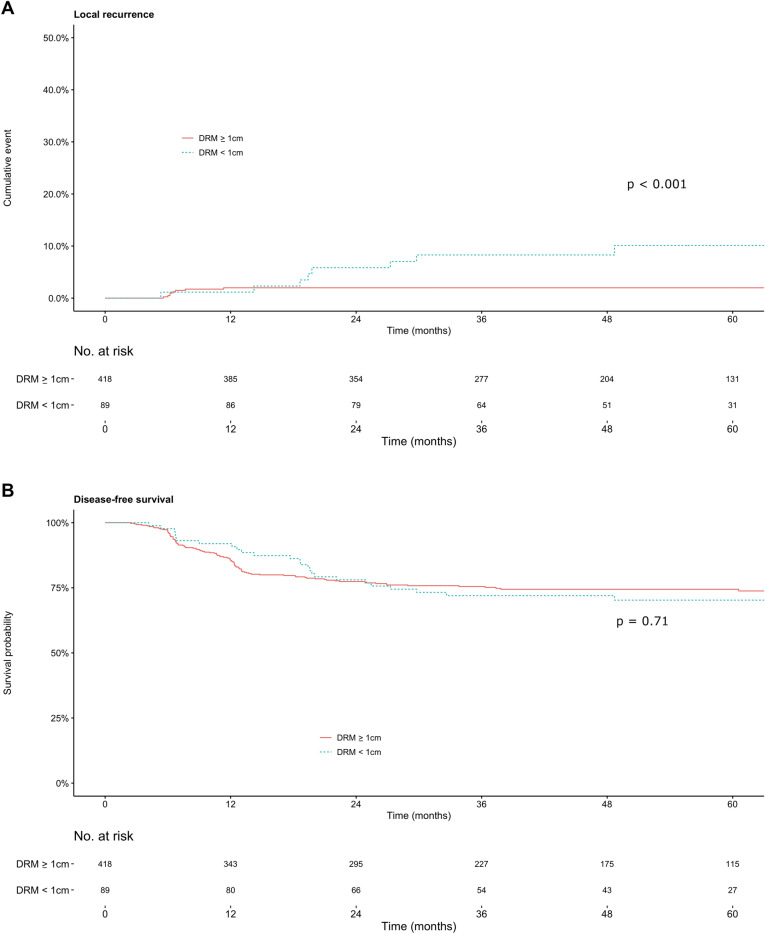
Cumulative incidence of local recurrence (**a**) and disease-free survival (**b**) in patients with locally advanced rectal cancer who underwent preoperative chemoradiotherapy followed by rectal excision.

The median follow-up period was 48.9 months (46.9 months in the ≥ 1 cm group versus 54.8 months in the < 1 cm group). Cumulative incidences of local recurrence at 3 years were 2% (95% confidence interval [CI], 0.6%–3.3%) for the ≥ 1 cm group and 8% (95% CI, 2.2%–14%) for the < 1 cm group (*P* < 0.001; Fig. 2A), indicating a significant difference. Cumulative incidences of distant metastasis at 3 years were 22.8% (95% CI, 18.6%–26.8%) for the ≥ 1 cm group and 23% (95% CI, 13.8%–31.8%) for the < 1 cm group (*P* = 0.80). The 5-year disease-free survival (DFS) rates were similar between the ≥ 1 cm and < 1 cm groups (74.4% vs. 70%; *P* = 0.71; Fig. 2B). Local recurrence was seen in the central region in six patients and in the pelvic sidewall in two patients in the < 1 cm group; three and five patients had local recurrence in the central region and pelvic sidewall, respectively, in the ≥ 1 cm group.

**Figure 3:**
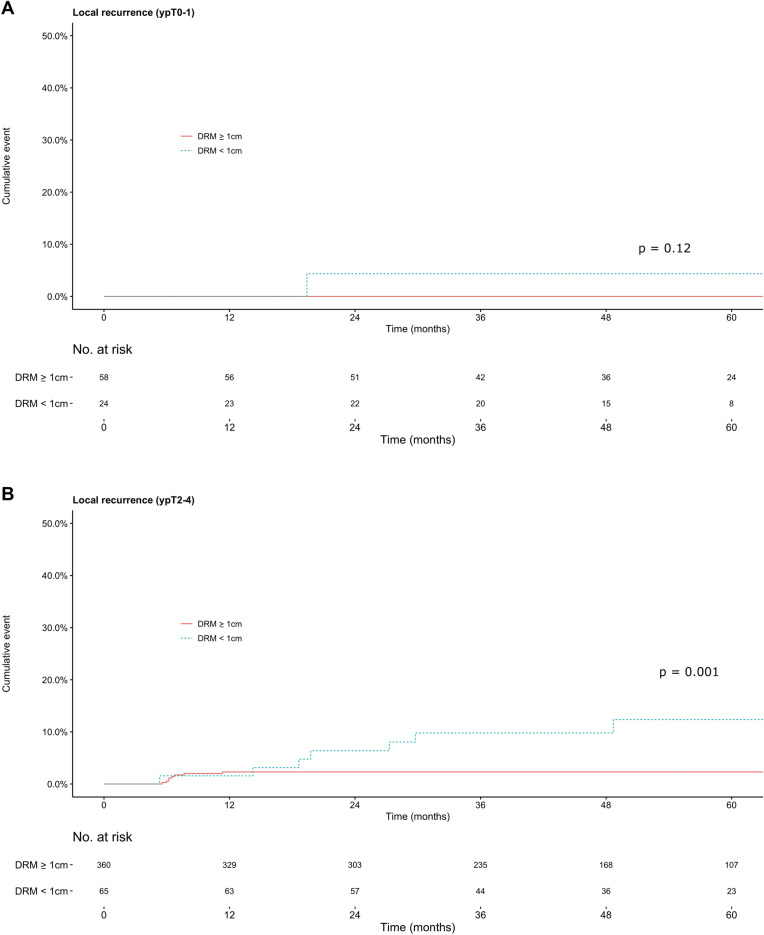
Cumulative incidence of local recurrence in patients with rectal cancer stages ypT0–1 (**a**) and ypT2–4 (**b**).

In the subgroup analysis, in the ypT0–1 group, the cumulative incidences of local recurrences at 3 years were 0% for the ≥ 1 cm group and 4% (95% CI, 0%–12.3%) for the < 1 cm group (*P* = 0.12; Fig. 3A). In the ypT2–4 group, the incidences were 2.3% (95% CI, 0.7%–3.9%) for the ≥ 1 cm group and 9.8% (95% CI, 2.0%–20.7%) for the < 1 cm group (*P* = 0.001; Fig. 3B).

The results of univariate and multivariable analyses of risk factors for local recurrence are listed in Table 3. Local recurrence was significantly associated with the DRM dimension (odds ratio [OR] = 4.57, 95% CI 1.48–14.5) and CRM status (OR = 4.77, 95% CI 1.35–15.3). In patients with ypT2–4, a short DRM (< 1 cm) was an independent risk factor for local recurrence (OR = 4.33, 95% CI 1.34–14.0; Supplemental Table S1). We could not find any significant risk factors associated with local recurrence in patients with ypT0–1 grade tumors. The results of risk factor analysis for local recurrence among patients with a negative CRM (> 1 mm) showed that a short DRM (< 1 cm) was a significant risk factor for local recurrence (OR = 5.15, 95% CI 1.38–20.4; Supplemental Table S2).

**Table 3:** Univariate and multivariable analyses of risk factors for local recurrence in patients with locally advanced rectal cancer who underwent preoperative chemoradiotherapy followed by rectal excision.

Characteristic	Univariate			Multivariable		
	OR	95% CI	*P*	OR	95% CI	*P*
Sex (male)	1.35	0.48, 4.35	0.60			
Age (> 60 years)	0.67	0.24, 1.82	0.40			
Tumor height (< 5 cm)	2.94	1.05, 9.46	0.048	1.84	0.56, 6.61	0.31
Clinical stage, T4	0.75	0.12, 2.74	0.71			
Clinical stage, N +	3.98	0.79, 72.4	0.21	4.75	0.90, 87.7	0.14
ypT3,4	1.10	0.39, 3.55	0.93			
ypN +	1.09	0.37, 2.99	0.94			
Histologic type (mucinous or signet-ring cell)	6.48	0.33, 43.6	0.10	10.4	0.49, 85.1	0.05
Positive CRM (≤ 1 mm)	4.74	1.44, 13.7	0.006	4.77	1.35, 15.3	0.01
DRM (< 1 cm)	5.06	1.81, 14.1	0.002	4.57	1.48, 14.5	0.008
Lymphovascular invasion	0.90	0.05, 4.63	> 0.9			
Venous invasion	1.92	0.29, 7.25	0.41			

*CRM* circumferential resection margin, *DRM* distal resection margin.

### Subanalysis

In patients who underwent sphincter-saving surgery (Supplemental Tables S3, S4), a short DRM (< 1 cm) (OR = 3.90, 95% CI 1.25–12.5; Supplemental Table S5 and CRM status (OR = 5.19, 95% CI 1.46–16.7) were independent risk factors for local recurrence on the multivariable analysis. In patients with tumor stages ypT2–4, a short DRM (< 1 cm) was an independent risk factor for local recurrence (OR = 3.66, 95% CI 1.11–12.1).

The subanalysis of the data from 482 patients without pathological lateral pelvic lymph node metastasis produced similar results (Supplemental Tables S6, S7, S8). The 3-year local recurrence rates were 1.8% and 7.2% in the ≥ 1 cm and < 1 cm groups, respectively (*P* < 0.001). The subgroup analysis revealed that a DRM of < 1 cm was not an independent risk factor for local recurrence in the ypT0–1 group. However, among patients with tumor stages ypT2–4, the cumulative 3-year incidences of local recurrence were 2.2% and 8.4% in the ≥ 1 cm and < 1 cm groups, respectively (*P* < 0.01).

The other subanalysis of data from 435 patients with tumor stages ypT1 − 4 showed similar results (Supplemental Tables S9, S10, S11). The 3-year local recurrence rates were 2.3% and 9.3% in the ≥ 1 cm and < 1 cm groups, respectively (*P* < 0.01). Among patients with tumor stages ypT1–2, the cumulative 3-year incidences of local recurrence were 1.5% and 11.5% in the ≥ 1 cm and < 1 cm groups, respectively (*P* = 0.07). However, in patients with tumor stages ypT3–4, the cumulative 3-year incidences of local recurrence were 2.5% and 7.7% in the ≥ 1 cm and < 1 cm groups, respectively (*P* = 0.03).

## Discussion

Although international guidelines recommend that a 1–2-cm DRM might be acceptable in patients with LARC^1, 2^, it is difficult to obtain a sufficient DRM for sphincter-saving surgery in patients with mid/low rectal cancer receiving preoperative CRT. In a systematic review that analyzed 5,574 patients with rectal cancer from 17 studies, a short DRM (< 1 cm) was observed in 948 (17.0%) patients^9^. In the Lyon R90-01 trial, 36.1% (43/119) of patients had a DRM of < 1.5 cm^10^. Among the patients with a suspected margin of < 1 cm after specimen extraction, we were unable to predict which of them had a risk of an adverse oncologic outcome. Even when the intraoperatively suspected DRM is not sufficient, it is technically challenging to perform additional rectal resection after a distal one. There are few published data on the impact of a short DRM on local recurrence in patients with LARC who underwent preoperative CRT, and the results are conflicting. Furthermore, very few investigators have directly evaluated the association between a short DRM and local recurrence, according to the ypT stage. We found that a short DRM (< 1 cm) was associated with local failure in patients with LARC receiving preoperative CRT; this was distinctly observed in patients with ypT2-4 tumors.

In the short DRM group (< 1 cm), over half of patients who developed local recurrence had a relapse in the central region, such as the presacral region, or at the anastomosis. The reasons for this are uncertain; however, some researchers have reported that residual cancer cells might be associated with local recurrence in the central pelvis, such as in the presacral region, or at the anastomosis within the radiation field^11–14^. These studies suggested that it might be related to the resection margin or tears at the tumor site. Upon microscopic analysis, tumor cells were noted to be scattered in nonuniform and unpredictable patterns after preoperative CRT^3^. Furthermore, tumor cells were present under normal-appearing mucosa in approximately 36–71% of cases, and the distance varied by up to 3–4 cm^3, 4^. They raised concerns that the distal margin could be invisible after preoperative CRT. Taken together, these findings indicate that a short DRM might be associated with local recurrence in the central region.

It is noteworthy that the impact of a short DRM (< 1 cm) on local recurrence was observed in patients with ypT2–4 stage disease. In contrast, such a short DRM was not a significant risk factor for local recurrence in patients with ypT0–1 stage tumors. We believe this difference arose from the various effects of CRT and different distal mural spreading patterns according to tumor stage. Some investigators have reported increased distal spread with increasing tumor stage^4, 15^. Shimada et al. reported that the maximum distances of distal spread were 4, 16, and 20 mm in tumor stages I, II, and III, respectively^15^. Smith et al. analyzed patients with LARC who underwent preoperative CRT and reported that the median and maximum microscopic tumor spread for ypT1 grade tumors were 0 mm and 4 mm, those for ypT2 were 2.5 mm and 9 mm, and those for ypT3 were 4 mm and 9 mm, respectively^4^. These results and those of our study reinforce the importance of achieving a sufficient DRM in patients suspected of having advanced ypT stage tumors.

To our knowledge, there are no guidelines on the indications for adjuvant treatment and treatment strategies for patients with a close or positive DRM after preoperative CRT and sphincter-preserving surgery. Recent guidelines have suggested that intraoperative radiation therapy might be considered for very close or positive margins after resection, as an additional boost^1, 16^. Kusters et al. reported the effectiveness of intraoperative radiotherapy for local control in patients with LARC^14^. Some investigators recommend adjuvant chemoradiation or salvage APR for patients with a pathologically invaded resection margin^17^. In cases of ypT2–4 stage tumors with a short DRM, more intensified combined treatment modalities, such as booster radiation, oxaliplatin-based chemotherapy, and closer follow-up, should be considered after multidisciplinary team meetings.

Other reports have recently analyzed the role of the DRM in patients with rectal cancer^18^. They demonstrated that the DRM did not influence oncologic outcomes, except in patients who had a poor response to neoadjuvant radiation. While our results show that a short DRM might influence local recurrence, especially in patients with ypT2–4 tumors, our study differed from the previous report in that we included all patients who received preoperative long-course CRT and excluded those with stage IV cancer. In addition, we analyzed the results based on ypT staging. Conversely, Kazi et al. included patients who had received long-course CRT, short-course radiation, or no preoperative treatment. They conducted subgroup analysis by tumor regression grade. They also showed that the DRM length predicted oncologic outcomes when the response to preoperative treatment was poor.

Pathologic CRM involvement is a well-known predictive factor for local recurrence, which could have influenced our results^19, 20^. Therefore, we analyzed independent risk factors for local recurrence with and without the CRM as a variable. Regardless of the pathologic CRM status, a short DRM (< 1 cm) was demonstrated to be a significant risk factor for local recurrence. This was also observed in the subgroup analysis of patients with ypT2–4 stage tumors.

This study had some limitations. It was retrospective, and bias was possible in terms of case selection. Despite this, our results were contrary to what was expected in terms of the characteristics of both groups. In detail, the local recurrence rate was higher in the short DRM (< 1 cm) group, which represents a favorable stage, than in the long DRM (≥ 1 cm) group. We did not analyze the results based on both intramural and mesorectal distal spread. Previous studies on DRM have described different methodologies to measure the resection margin status^9^, and some have focused on whole-mount histopathology analyses to evaluate the DRM^15, 21, 22^. However, our routine histopathology examinations were performed according to the international standard reporting form. Therefore, our results might reflect real clinical practices. To the best of our knowledge, this study includes the largest reported number of patients (over 500) with LARC who underwent preoperative CRT. Moreover, we adopted strict criteria for neoadjuvant treatments and analyzed the results based on ypT staging.

In conclusion, a DRM of < 1 cm might lead to local treatment failure in patients with LARC who underwent preoperative CRT. This was clearly observed in patients with ypT2–4 tumors. Adjuvant treatment agreed upon through multidisciplinary team conferences should be considered in patients with ypT2–4 grade tumors and a short DRM.

## Methods

### Patients

This study was approved by our institutional review board (KNUCH 2020–04-034). In accordance with the institution’s guidelines and regulations for retrospective studies, the institutional review board waived the requirement for informed consent (institutional review board of Kyungpook National University Chilgok Hospital). We performed this study in accordance with the Declaration of Helsinki.

We analyzed data from our hospital’s prospectively collected colorectal cancer registry for patients treated between January 2011 and March 2017. We reviewed patients with rectal cancers located within 12 cm from the anal verge during initial imaging and who underwent neoadjuvant CRT. Patients with stage IV tumors were excluded. Tumor location was assessed using digital examination, rigid sigmoidoscopy, or pelvic MRI. All patients underwent physical examinations, chest, abdominal, and pelvic computed tomography (CT), and pelvic MRI. After neoadjuvant CRT, restaging was performed 6–7 weeks later via pelvic MRI and chest, abdominal, and pelvic CT.

### Neoadjuvant treatments

Neoadjuvant CRT was recommended for patients with cT tumors of any N1–2 stage or cT3–4N0 disease. Patients underwent concurrent CRT, comprising a total irradiation dose of 45–50.4 Gy delivered at 2 Gy per day, 5 days per week, for 5 weeks, and concurrent chemotherapy included a continuous infusion of 5-fluorouracil (5-FU)/leucovorin or oral capecitabine. During the study period, the most common chemotherapeutic regimens consisted of a 5-FU (425 mg/m^2^) intravenous bolus plus a leucovorin (20 mg/m^2^) intravenous bolus for 4 days during weeks 1 and 5 of radiation or oral capecitabine (825 mg/m^2^) twice daily, 5 days per week, for 5 weeks. Surgery was scheduled 7–9 weeks after the completion of radiation.

### Surgical procedures

Patients underwent mechanical bowel preparation before surgery. A standard total mesorectal excision was performed as described previously^23, 24^. We performed a high ligation of the inferior mesenteric artery, medial-to-lateral mobilization of the left colon, complete mobilization of the splenic flexure, and sharp dissection of the pelvis with a nerve-sparing technique. Selective lateral pelvic lymph node dissection was performed in patients with suspected lateral pelvic node metastases identified on initial imaging^25^. Sphincter-saving was performed in all patients, except when the levator ani muscle had been invaded by the tumor. Our policy was a DRM of > 2 cm for upper and mid rectal cancers. For low rectal cancers, we attempted to achieve a distal clearance margin of ≥ 0.5 cm for sphincter preservation. Anastomosis was performed using either double-stapled, end-to-end, or hand-sewn techniques, except for APRs. All procedures were performed by four experienced surgeons (G.-S.C., J.S.P., S.Y.P., and H.J.K.) who had each performed over 100 colorectal cancer surgeries per year.

### Histopathology

The surgical specimens were first inspected externally to locate the tumor, and the presence of any obvious perforation was recorded intraoperatively^26^. The specimens were then transferred to the pathology department and examined by two specialist pathologists (G.S.Y. and A.N.S.) with > 10 years of experience in colorectal cancer pathology. The non-peritonealized surface of the specimen was marked with green ink to enable subsequent identification of the CRM. After this, the specimen was opened along the anterior aspect and fixed in a large volume of 10% neutral-buffered formalin overnight. Subsequently, the tumor size and distance of the tumor from the proximal and distal resection margins were measured. The DRM was defined as the distance from the lower edge of the primary rectal cancer (or residual ulcer or scar at the original tumor site in patients showing complete tumor regression) to the resection margin of the specimen. In cases of stapled anastomoses, donut rings were not included in the measurement but were examined microscopically to determine the involvement of any viable tumor cells. If proximal or distal resection margins of ≤ 1.5 cm were detected visually, appropriate blocks were selected to cover the closest approximation to those margins and then evaluated microscopically. The CRM was defined as the closest distance from the outermost part of the viable tumor cells to the inked resected specimen. The CRM was measured using a microscope graticule or ruler, and CRM negativity was defined as a distance of > 1 mm^27^. The pathologic stage of each tumor was assigned following the guidelines from the 7th edition of the American Joint Committee on Cancer staging manual for colorectal cancers^28^. Regression of the primary tumor in response to neoadjuvant CRT was assessed based on the TRG, as described by Rodel et al.^29^.

### Adjuvant chemotherapy

The most common adjuvant chemotherapeutic regimens were four more cycles of fluorouracil and leucovorin (fluorouracil 400–425 mg/m^2^ per day plus leucovorin 20 mg/m^2^ per day on days 1–5, every 4 weeks) or eight more cycles of modified FOLFOX 6 (oxaliplatin 85 mg/m^2^ on day 1, leucovorin 400 mg total dose over 2 h on day 1, fluorouracil 400 mg/m^2^ bolus on day 1, followed by 2,400 mg/m^2^ over 46 h, every 2 weeks).

### Follow-up

Patients were followed up via clinical examination, blood assays for carcinoembryonic antigen, and chest, abdominal, and pelvic CT every 3 months in the first 2 years and every 6 months thereafter. Patients routinely underwent colonoscopy in the first and fourth years after surgery. Local recurrence was defined as recurrent disease within the true pelvis with or without distant metastases and was classified into central (anastomotic, anterior, presacral, and perineal) and lateral (pelvic sidewall) recurrence^11, 12^. Distant recurrence was defined as recurrence beyond the locoregional area.

### Statistical analysis

Continuous variables were first tested for normality (Shapiro–Wilk Normality test). The two groups based on DRM (≥ 1 cm and < 1 cm) were compared using two-sample Student’s *t* tests or Kruskal–Wallis Rank Sum tests. Categorical variables were assessed using the chi-square test or Fisher’s exact test. Logistic regression was applied to multivariable analysis to identify independent risk factors for local recurrence. Variables with a *P*-value of < 0.2 in the univariate analysis were selected for the multivariable analysis. DFS was defined as the period from the date of initial surgery to the date of disease recurrence or death. The time to local recurrence was calculated from the date of initial surgery to the date that local recurrence was noted; patients with no recurrence were censored at the date of the last follow-up or at death. Patient survival was estimated using the Kaplan–Meier method, and survival curves were compared using a log-rank test. We conducted a subanalysis using the following criteria: (1) patients who underwent sphincter-saving surgery were included, (2) patients with pathologic lateral pelvic lymph node metastasis were excluded, and (3) patients with a pathologic complete response were excluded. All analyses were conducted using the R: A Language and Environment for Statistical Computing, Version 4.0.1 (R Core Team, R Foundation for Statistical Computing, Vienna, Austria, 2020; https://www.R-project.org), and *p* values < 0.05 were considered statistically significant.

### Ethics approval

This study was approved by the institutional review board (KNUCH 2020–04-034).

## Supplementary Information

Supplementary Information.

## Data Availability

The datasets used and analyzed during the current study are available from the corresponding author on reasonable request.
