# Supplementary material for: Impact of the distal resection margin on local recurrence after neoadjuvant chemoradiation and rectal excision for locally advanced rectal cancer
Source: Sci Rep. 2021 Nov 25;11:22943. doi: 10.1038/s41598-021-02438-1 (PMC8617265; doi:10.1038/s41598-021-02438-1)
Supplement: Supplementary file 1 — Supplementary Information. [file 41598_2021_2438_MOESM1_ESM.pdf]

Impact of the Distal Resection Margin on Local Recurrence After Neoadjuvant Chemoradiation and Rectal Excision for Locally Advanced Rectal Cancer

Seung Ho Song, M.D.[1], Jun Seok Park, M.D.[1*], Gyu-Seog Choi, M.D.[1*], An Na Seo, M.D.[2], Soo Yeun Park, M.D.[1], Hye Jin Kim, M.D.[1], Sung-Min Lee, M.D.[1], Ghilsuk Yoon, M.D.[2]

[1]Colorectal Cancer Center, Kyungpook National University Chilgok Hospital, School of Medicine, Kyungpook National University, Daegu, Republic of Korea

[2]Department of Pathology, Kyungpook National University Chilgok Hospital, School of Medicine, Kyungpook National University, Daegu, Republic of Korea

Supplementary Information Listing:

Supplementary Table S1, S2, S3, S4, S5, S6, S7, S8, S9, S10, and Table S11

* Corresponding authors (contributed equally as corresponding authors)

Jun Seok Park, M.D.

Professor, Colorectal Cancer Center,

Kyungpook National University Chilgok Hospital,

807 Hogukro, Buk-gu, Daegu 40414, Korea

Phone number: +82-53-200-2772

Fax: +82-53-200-2027

E-mail: parkjs0802@knu.ac.kr

Gyu-Seog Choi, M.D.

Professor, Colorectal Cancer Center,

Kyungpook National University Chilgok Hospital,

807 Hogukro, Buk-gu, Daegu 41404, Korea

Phone number: +82-53-200-2166

Fax: +82-53-200-2027

E-mail: kyuschoi@mail.knu.ac.kr

Supplementary Table S1. Univariate and multivariable analyses of risk factors for local recurrence in patients with locally advanced rectal cancer who underwent preoperative chemoradiotherapy followed by rectal excision (ypT2-4). CRM, circumferential resection margin. DRM, distal resection margin.

	Univariate			Multivariable		
**Characteristic**	**OR**	**95% CI**	***p***	**OR**	**95% CI**	***p***
Sex (male)	1.31	0.46, 4.26	0.61			
Age (> 60 years)	0.76	0.26, 2.14	0.63			
Tumor height (< 5 cm)	3.16	1.10, 10.3	0.04	2.08	0.61, 7.56	0.20
Clinical stage, T4	0.71	0.11, 2.64	0.70			
Clinical stage, N+	3.50	0.69, 63.9	0.25	4.26	0.80, 79.0	0.20
ypT3,4	0.72	0.24, 2.65	0.63			
ypN+	1.10	0.06, 5.82	>0.9			
Histologic type (mucinous or signet-ring cell)	5.79	0.29, 39.2	0.12	8.11	0.38, 65.3	0.08
Positive CRM (≤ 1 mm)	3.10	0.83, 9.53	0.06	2.93	0.73, 10.0	0.10
DRM (< 1 cm)	5.31	1.80, 15.3	0.002	4.33	1.34, 14.0	0.01
Lymphovascular invasion	0.79	0.04, 4.12	0.81			
Venous invasion	1.82	0.28, 6.96	0.43			

Supplementary Table S2. Univariate and multivariable analyses of risk factors for local recurrence in patients with locally advanced rectal cancer who underwent preoperative chemoradiotherapy followed by rectal excision (circumferential resection margin free). DRM, distal resection margin.

	Univariate			Multivariable		
**Characteristic**	**OR**	**95% CI**	***p***	**OR**	**95% CI**	***p***
Sex (male)	1.45	0.41, 6.70	0.6			
Age (> 60 years)	0.48	0.12, 1.62	0.2	0.44	0.11, 1.53	0.2
Tumor height (< 5 cm)	2.56	0.76, 9.88	0.14	1.70	0.44, 7.43	0.5
Clinical stage, T4	0.00		>0.9			
Clinical stage, N+	0.00		>0.9			
ypT3,4	1.53	0.43, 7.04	0.5			
ypN+	1.11	0.29, 3.74	0.9			
Histologic type (mucinous or signet-ring cell)	8.86	0.44, 62.1	0.056	14.9	0.69, 12.8	0.076
DRM (< 1 cm)	5.97	1.75, 21.2	0.004	5.15	1.38, 20.4	0.015
Lymphovascular invasion	0.00		>0.9			
Venous invasion	1.50	0.08, 8.26	0.7			

Supplementary Table S3. Patient characteristics in patients underwent sphincter-saving surgery. DRM, distal resection margin.

	DRM ≥ 1 cm (n = 391)	DRM < 1 cm (n = 89)	*p*
Age, years	62.0 (55.0–71.0)	60.0 (53.0–69.0)	0.19
Sex			0.20
Male	237 (60.6%)	61 (68.5%)	
Female	154 (39.4%)	28 (31.5%)	
Tumor height, cm	6.0 (4.0–8.0)	2.5 (2.0–4.2)	<0.001
Clinical T stage			<0.001
T2	3 (0.8%)	5 (5.6%)	
T3	322 (82.4%)	79 (88.8%)	
T4	66 (16.9%)	5 (5.6%)	
Clinical N stage			0.13
N0	71 (18.2%)	23 (25.8%)	
N+	320 (81.8%)	66 (74.2%)	

Supplementary Table S4. Operative and pathologic findings in patients underwent sphincter-saving surgery. DRM, distal resection margin. LAR, low anterior resection. ISR, intersphincteric resection. LPND, lateral pelvic lymph node dissection. CRM, circumferential resection margin. LPN, lateral pelvic lymph node.

	DRM ≥ 1 cm (n = 391)	DRM < 1 cm (n = 89)	*p*
Type of operation			<0.001
LAR	286 (73.1%)	35 (39.3%)	
ISR	105 (26.9%)	54 (60.7%)	
LPND	65 (16.6%)	11 (12.4%)	0.40
Tumor size, cm	3.2 (2.0–4.5)	2.5 (1.8–3.3)	<0.001
Lymphovascular invasion	27 (6.9%)	3 (3.4%)	0.32
Venous invasion	28 (7.2%)	4 (4.5%)	0.50
Pathologic stage			<0.001
ypT0N0	42 (10.7%)	21 (23.6%)	
ypT0N+	6 (1.5%)	-	
I	54 (13.8%)	24 (27.0%)	
II	140 (35.8%)	30 (33.7%)	
III	149 (38.1%)	14 (15.7%)	
CRM, positive (≤ 1 mm)	31 (7.9%)	8 (9.0%)	0.91
Pathologic LPN positive	19/65	2/11	0.69
Tumor regression grade			0.003
0 (no regression)	5 (1.3%)	2 (2.3%)	
1 (minor regression)	48 (12.8%)	8 (9.2%)	
2 (moderate regression)	136 (36.3%)	15 (17.2%)	
3 (good regression)	138 (36.8%)	41 (47.1%)	
4 (total regression)	48 (12.8%)	21 (24.1%)	

Supplementary Table S5. Univariate and multivariable analysis of risk factors for local recurrence in patients with locally advanced rectal cancer who underwent preoperative chemoradiotherapy followed by sphincter-saving surgery. CRM, circumferential resection margin. DRM, distal resection margin.

	Univariate			Multivariable		
**Characteristic**	**OR**	**95% CI**	***p***	**OR**	**95% CI**	***p***
Sex (male)	1.36	0.48, 4.37	0.60			
Age (> 60 years)	0.68	0.24, 1.86	0.50			
Tumor height (< 5 cm)	3.35	1.20, 10.8	0.027	2.07	0.62, 7.50	0.20
Clinical stage, T4	0.82	0.13, 3.01	0.80			
Clinical stage, N+	3.76	0.75, 68.4	0.20	4.42	0.83, 81.9	0.20
ypT3,4	1.09	0.39, 3.52	0.90			
ypN+	1.11	0.37, 3.04	0.70			
Histologic type (mucinous or signet-ring cell)	6.12	0.31, 41.2	0.11	10.1	0.48, 80.7	0.052
Positive CRM (≤ 1 mm)	5.75	1.73, 16.8	0.002	5.19	1.46, 16.7	0.007
DRM (< 1 cm)	4.73	1.69, 13.2	0.003	3.90	1.25, 12.5	0.019
Lymphovascular invasion	1.00	0.05, 5.21	>0.9			
Venous invasion	2.07	0.32, 7.86	0.40			

Supplementary Table S6. Patient characteristics in patients without pathologic lateral pelvic lymph node metastasis. DRM, distal resection margin.

	DRM ≥ 1 cm (n = 395)	DRM < 1 cm (n = 87)	*p*
Age, years	62.0 (55.0–71.0)	60.0 (53.5–69.0)	0.20
Sex			0.15
Male	237 (60.0%)	60 (69.0%)	
Female	158 (40.0%)	27 (31.0%)	
Tumor height, cm	6.0 (4.0–8.0)	2.5 (2.0–4.2)	<0.001
Clinical T stage			<0.001
T2	3 (0.8%)	5 (5.7%)	
T3	319 (80.8%)	77 (88.5%)	
T4	73 (18.5%)	5 (5.7%)	
Clinical N stage			0.24
N0	79 (20.0%)	23 (26.4%)	
N+	316 (80.0%)	64 (73.6%)	

Supplementary Table S7. Operative and pathologic findings in patients without pathologic lateral pelvic lymph node metastasis. DRM, distal resection margin. LAR, low anterior resection. ISR, intersphincteric resection. APR, abdominoperineal resection. LPND, lateral pelvic lymph node dissection. CRM, circumferential resection margin.

	DRM ≥ 1 cm (n = 395)	DRM < 1 cm (n = 87)	*p*
Type of operation			<0.001
LAR	277 (70.1%)	35 (40.2%)	
ISR	95 (24.1%)	52 (59.8%)	
APR	23 (5.9%)	-	
LPND	49 (12.4%)	9 (10.3%)	0.72
Tumor size, cm	3.2 (2.0–4.5)	2.5 (1.8–3.3)	<0.001
Lymphovascular invasion	27 (6.8%)	3 (3.4%)	0.35
Venous invasion	27 (6.8%)	4 (4.6%)	0.60
Pathologic stage			<0.001
ypT0N0	45 (11.4%)	21 (24.1%)	
ypT0N+	5 (1.3%)	-	
I	60 (15.2%)	24 (27.6%)	
II	146 (37.0%)	30 (34.5%)	
III	139 (35.2%)	12 (13.8%)	
CRM, positive (≤ 1 mm)	37 (9.4%)	7 (8.0%)	0.86
Tumor regression grade			0.002
0 (no regression)	3 (0.8%)	2 (2.4%)	
1 (minor regression)	49 (12.9%)	8 (9.4%)	
2 (moderate regression)	135 (35.6%)	14 (16.5%)	
3 (good regression)	142 (37.5%)	40 (47.1%)	
4 (total regression)	50 (13.2%)	21 (24.7%)	

Supplementary Table S8. Univariate and multivariable analysis of risk factors for local recurrence in patients with locally advanced rectal cancer who underwent preoperative chemoradiotherapy followed by rectal excision (without pathologic lateral pelvic lymph node metastasis). CRM, circumferential resection margin. DRM, distal resection margin.

	Univariate			Multivariable		
**Characteristic**	**OR**	**95% CI**	***p***	**OR**	**95% CI**	***p***
Sex (male)	1.12	0.38, 3.71	0.80			
Age (> 60 years)	0.65	0.21, 1.89	0.40			
Tumor height (< 5 cm)	2.54	0.86, 8.38	0.10	1.29	0.35, 4.97	0.70
Clinical stage, T4	0.86	0.13, 3.24	0.80			
Clinical stage, N+	3.58	0.70, 65.4	0.20			
ypT3,4	0.92	0.31, 3.03	0.90			
ypN+	0.83	0.23, 2.53	0.80			
Positive CRM (≤ 1 mm)	6.11	1.80, 18.6	0.002	6.38	1.75, 21.7	0.003
DRM (< 1 cm)	4.85	1.62, 14.5	0.004	5.10	1.46, 18.6	0.011
Lymphovascular invasion	1.16	0.06, 6.16	>0.9			
Venous invasion	2.52	0.38, 9.83	0.20			

Supplementary Table S9. Patient characteristics in patients with tumor stages ypT1-4. DRM, distal resection margin.

	DRM ≥ 1 cm (n = 367)	DRM < 1 cm (n = 68)	*p*
Age, years	62.0 (55.0–71.0)	60.0 (53.5–69.0)	0.25
Sex			0.18
Male	219 (59.7%)	47 (69.1%)	
Female	148 (40.3%)	21 (30.9%)	
Tumor height, cm	6.0 (4.0–8.0)	2.5 (2.0–4.5)	<0.001
Clinical T stage			<0.001
T2	3 (0.8%)	4 (5.9%)	
T3	292 (79.6%)	59 (86.8%)	
T4	72 (19.6%)	5 (7.4%)	
Clinical N stage			0.29
N0	68 (18.5%)	17 (25.0%)	
N+	299 (81.5%)	51 (75.0%)	

Supplementary Table S10. Operative and pathologic findings in patients with tumor stages ypT1-4. DRM, distal resection margin. LAR, low anterior resection. ISR, intersphincteric resection. APR, abdominoperineal resection. LPND, lateral pelvic lymph node dissection. CRM, circumferential resection margin. LPN, lateral pelvic lymph node.

	DRM ≥ 1 cm (n = 367)	DRM < 1 cm (n = 68)	*p*
Type of operation			<0.001
LAR	259 (70.6%)	28 (41.2%)	
ISR	84 (22.9%)	40 (58.8%)	
APR	23 (6.3%)	-	
LPND	62 (16.9%)	9 (13.2%)	0.57
Tumor size, cm	3.4 (2.2–4.5)	2.7 (2.0–3.5)	<0.001
Lymphovascular invasion	32 (8.7%)	3 (4.4%)	0.34
Venous invasion	31 (8.4%)	3 (4.4%)	0.37
Pathologic stage			<0.001
I	60 (16.3%)	24 (35.3%)	
II	147 (40.1%)	30 (44.1%)	
III	160 (43.6%)	14 (20.6%)	
CRM, positive (≤ 1 mm)	40 (10.9%)	7 (10.3%)	0.91
Pathologic LPN positive	22/62	2/9	0.68
Tumor regression grade			0.03
0 (no regression)	6 (1.7%)	2 (3.0%)	
1 (minor regression)	52 (14.8%)	8 (12.1%)	
2 (moderate regression)	144 (41.0%)	15 (22.7%)	
3 (good regression)	149 (42.5%)	41 (62.1%)	

Supplementary Table S11. Univariate and multivariable analysis of risk factors for local recurrence in patients with locally advanced rectal cancer who underwent preoperative chemoradiotherapy followed by rectal excision (patients with tumor stages ypT1-4). CRM, circumferential resection margin. DRM, distal resection margin.

	Univariate			Multivariable		
**Characteristic**	**OR**	**95% CI**	***p***	**OR**	**95% CI**	***p***
Sex (male)	1.28	0.45, 4.17	0.70			
Age (> 60 years)	0.76	0.26, 2.15	0.60			
Tumor height (< 5 cm)	3.09	1.08, 10.1	0.04	2.08	0.62, 7.51	0.20
Clinical stage, T4	0.71	0.11, 2.63	0.70			
Clinical stage, N+	3.50	0.69, 63.9	0.20	4.45	0.83, 82.4	0.20
ypT3,4	0.80	0.27, 2.95	0.70			
ypN+	1.00	0.33, 2.82	0.90			
Histologic type (mucinous or signet-ring cell)	5.93	0.30, 40.1	0.11	8.35	0.39, 67.3	0.08
Positive CRM (≤ 1 mm)	3.19	0.85, 9.79	0.056	3.03	0.76, 10.3	0.09
DRM (< 1 cm)	5.15	1.75, 14.9	0.002	3.90	1.25, 12.5	0.012
Lymphovascular invasion	0.81	0.04, 4.23	0.80			
Venous invasion	2.78	0.42, 7.15	0.40			
